# Novel Treatment for a Completely Extruded Talus

**DOI:** 10.31486/toj.24.0047

**Published:** 2024

**Authors:** Basel Taha, Colin Carroll, Rick Gehlert

**Affiliations:** Department of Orthopedics, University of New Mexico School of Medicine, Albuquerque, NM

**Keywords:** *Arthrodesis*, *multiple trauma*, *musculoskeletal pain*, *orthopedic procedures*, *talus*

## Abstract

**Background:** Complete talar extrusion is a rare injury that is typically caused by high-energy impact. Treatment for a completely extruded talus is limited and has variable outcomes and complications. Tibiocalcaneal arthrodesis is one of the best treatments for restoring stability and reducing pain in the affected limb.

**Case Report:** A 52-year-old male had complete talar extrusion after a pedestrian vs vehicle accident, and his recovery was complicated by wound dehiscence and recurrent infections of the ankle. Three years after his original injury, the patient was treated with a tibiocalcaneal arthrodesis with tantalum metal cone spacer, autologous bone grafting with tibial reamer irrigator aspirator, and retrograde hindfoot nail. The fusion healed well without signs of nonunion. Following wound healing and recovery, the patient was able to ambulate without any assistive devices.

**Conclusion:** To our knowledge, our case is the first report of the use of a metal spacer in conjunction with autologous bone grafting multiple years after an initial complete talar extrusion injury. The patient's novel treatment resulted in good postoperative outcomes, including significant improvement in pain, ankle stability, and independent ambulation.

## INTRODUCTION

Complete talar extrusion—total dislocation of the talus—is rare, typically occurs from a high-energy trauma, and can be challenging to manage.^1^ If the energy from the injury is significant enough, the talus can break through the talar ligaments and completely expel from the body.^1,2^ The talus has no muscle attachments, and more than 60% of the talus is covered by hyaline articular cartilage and ligamentous attachments, leaving a limited area for blood supply.^1,3^ After significant trauma, the lack of circulation and soft tissue in this area increases the risk of infection and nonhealing in patients with talar extrusion.^4,5^

Treatment for talar extrusion is variable based on the availability of the talus. A missing talus raises concerns for ankle stability and limb functionality. Surgical options for an extruded talus include amputation, open reduction and internal fixation, and tibiocalcaneal arthrodesis with or without talectomy.^1^ Early talectomy and tibiocalcaneal arthrodesis were previously recommended to lower the risk of avascular necrosis and infection.^5,6^ A review published in 2015, however, suggests that talectomy is an unfavorable option, associated with poor functional outcomes and a high risk of complications.^1^ A complete talar extrusion with loss of the entire talus leaves a void that needs to be filled and increases the risk of ipsilateral leg shortening.^5,7^ The use of tibiocalcaneal arthrodesis with prosthetics or allograft has been reported in the literature.^8-13^ However, to our knowledge, the use of a metal spacer in conjunction with autologous bone grafting multiple years after initial injury has not been documented.

## CASE REPORT

A 52-year-old male with no significant medical history was involved in a pedestrian vs vehicle accident and presented to a level 1 trauma center with multiple traumatic injuries and a Glasgow Coma Scale score of 3. His orthopedic injuries included a right complete talar extrusion, right hip dislocation with a posterior wall fracture, and multiple nonoperative spinal injuries. The patient's talus was not present on arrival and was likely left at the site of the accident.

**Figure 1.**
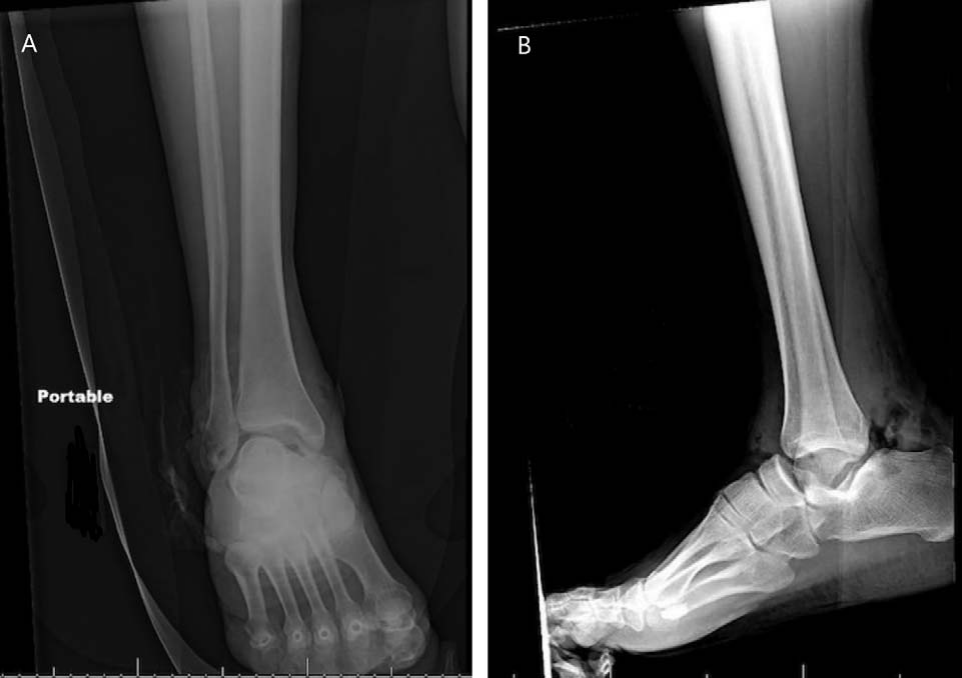
(A) Anteroposterior and (B) lateral emergency department x-rays of the patient's right ankle show complete talar extrusion with soft tissue gas secondary to an open talar extrusion.

The patient had a 6-cm laceration to the posteromedial aspect of the ankle, with noted deformity to the ankle. Radiographs revealed a complete talar extrusion (Figure 1). He had Dopplerable pedal pulses; motor and sensory examination was limited because the patient was intubated and sedated. The patient's wound was irrigated and closed, and he was placed in a short leg splint. The right hip dislocation was reduced, and femoral traction was placed because of hip instability.

Two days later, the patient was taken to the operating room for ankle spanning external fixator placement, irrigation and debridement of the right ankle, and placement of tobramycin- and vancomycin-infused polymethylmethacrylate beads.

**Figure 2.**
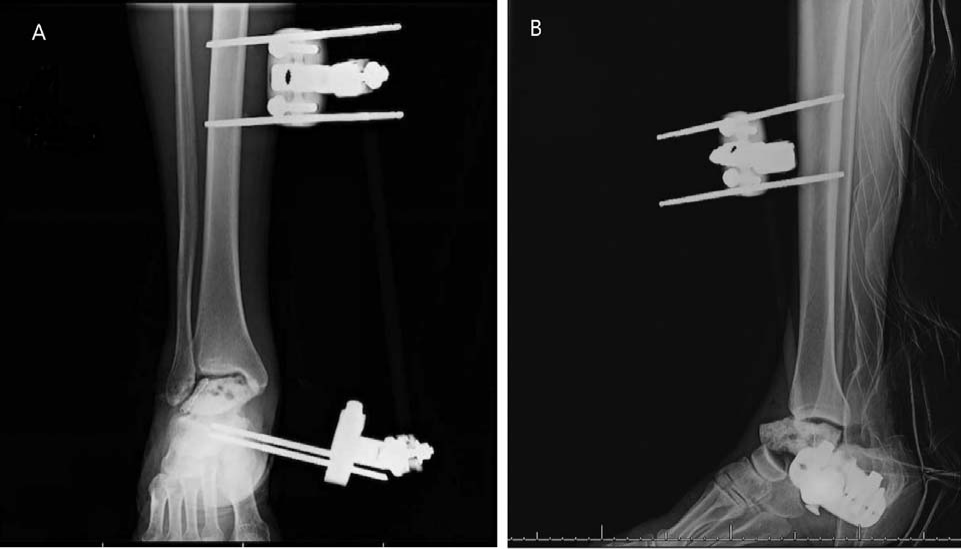
(A) Anteroposterior and (B) lateral x-rays of the patient's right ankle after cement spacer placement and external fixator adjustment.

A second irrigation and debridement of the right ankle was completed 3 days later with insertion of an antibiotic cement spacer with external fixator adjustment (Figure 2). The right posterior wall fracture from the hip dislocation was also addressed at that time with open reduction and internal fixation.

Physical examination performed after extubation revealed intact motor function and sensation of his right lower extremity. The patient was discharged 9 days later.

The patient presented to his follow-up appointments as scheduled, and his medial ankle wound was healing well until his 9-week postoperative visit when a small area of wound dehiscence was found. He was admitted for external fixator removal, irrigation and debridement, antibiotic cement spacer exchange, and wound vacuum-assisted closure application.

The ankle had wound healing issues and persistent drainage postoperatively that required repeat irrigation and debridement procedures. The wound culture grew polymicrobial bacteria. Infectious Diseases recommended an antibiotic regimen of 2 g intravenous (IV) cefepime 2 times daily and 500 mg oral metronidazole 3 times daily. Plastic Surgery was consulted for recommendations on wound coverage. Reverse sural flap procedure was completed for skin coverage. The patient remained in the hospital and required a second sural flap procedure because of compromise of the distal aspect of the sural flap. He was discharged after 35 days.

**Figure 3.**
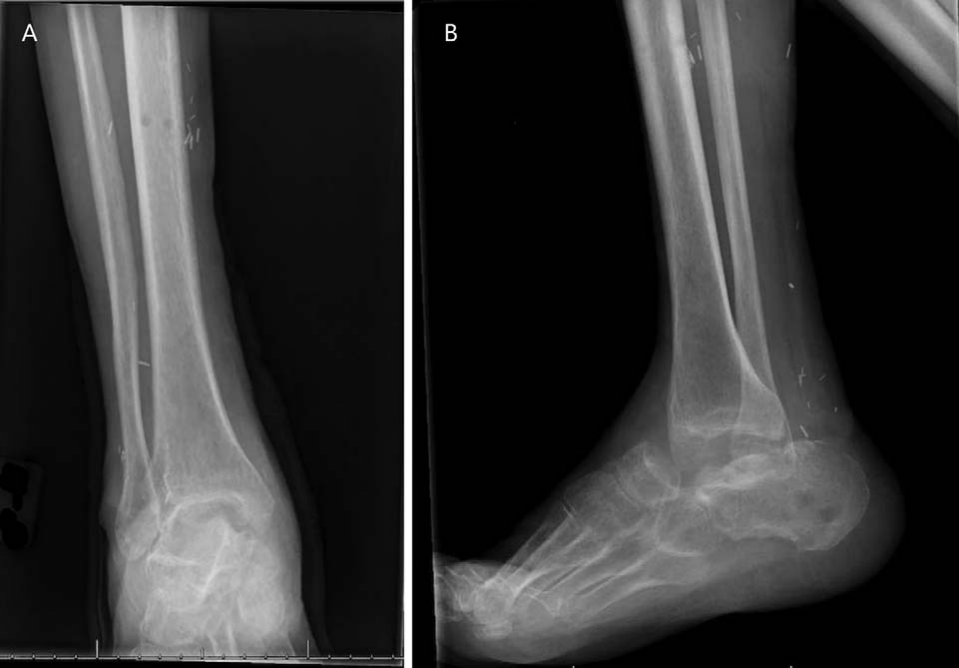
(A) Anteroposterior and (B) lateral x-rays of the patient's right ankle after antibiotic cement spacer removal.

After discharge, the orthopedic, infectious diseases, and plastic surgery outpatient clinics monitored the patient's wound. He remained on the same regimen of self-administered IV antibiotics and daily wound care for 1 week postdischarge. Six months after discharge, he developed right ankle septic arthritis with methicillin-sensitive *Staphylococcus aureus* and was readmitted for repeat irrigation and debridement and antibiotic cement spacer removal (Figure 3). The patient's antibiotic regimen during his hospital stay included vancomycin, cefepime, metronidazole, and cefazolin. He was discharged non-weight-bearing on his right lower extremity and with the recommendation from Infectious Diseases of 2 g cefazolin IV 3 times daily. After 2 weeks, Infectious Diseases switched this prescription to 100 mg oral doxycycline twice daily. The patient's laboratory values normalized, his wound healed, and he was taken off antibiotics 6 weeks after discharge. He remained non-weight-bearing on the right lower extremity until he was seen for avascular necrosis of his right femoral head from his prior hip dislocation. He underwent a right total hip replacement 8 months after his most recent hospital admission.

The patient used a cane for ambulation after his total hip replacement, but he had considerable ankle instability and pain. An ankle-foot orthosis brace provided limited relief of his symptoms. Five months after his total hip replacement and 3 years after his original injury, the patient underwent a tibiocalcaneal arthrodesis with retrograde hindfoot nail. This shared decision was made with the goal to improve the patient's ankle instability and leg length discrepancy that were the reasons for his pain and limited mobility.

**Figure 4.**
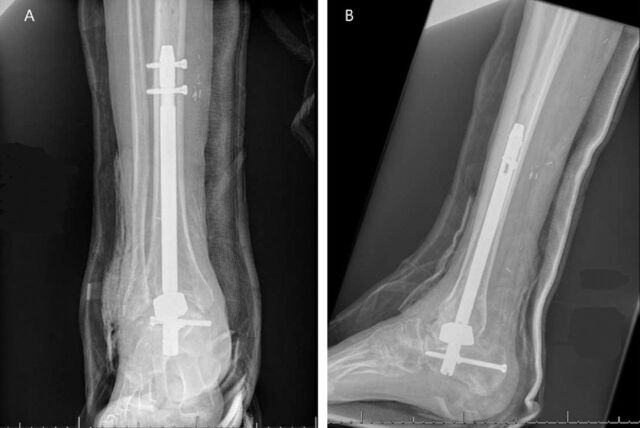
(A) Anteroposterior and (B) lateral x-rays of the patient's right ankle after hindfoot nail fusion with a tantalum metal spacer.

A tantalum metal cone used in total knee arthroplasty procedures was used as a spacer to help restore height to the patient's hindfoot and to provide a surface for fusion between the calcaneus and tibia. The tantalum cone was cut twice with 2 separate diamond wheels to allow the ankle to get to a neutral 0^o^ angle in terms of dorsiflexion and plantar flexion. A retrograde hindfoot nail (Zimmer Biomet) was placed with the use of a reamer irrigator aspirator with which 20 cm^3^ of bone graft was obtained. The nail was directed through the tantalum spacer and compressed at the level of the ankle (Figure 4). Bone graft from the reamer irrigator aspirator was packed around the tantalum spacer.

**Figure 5.**
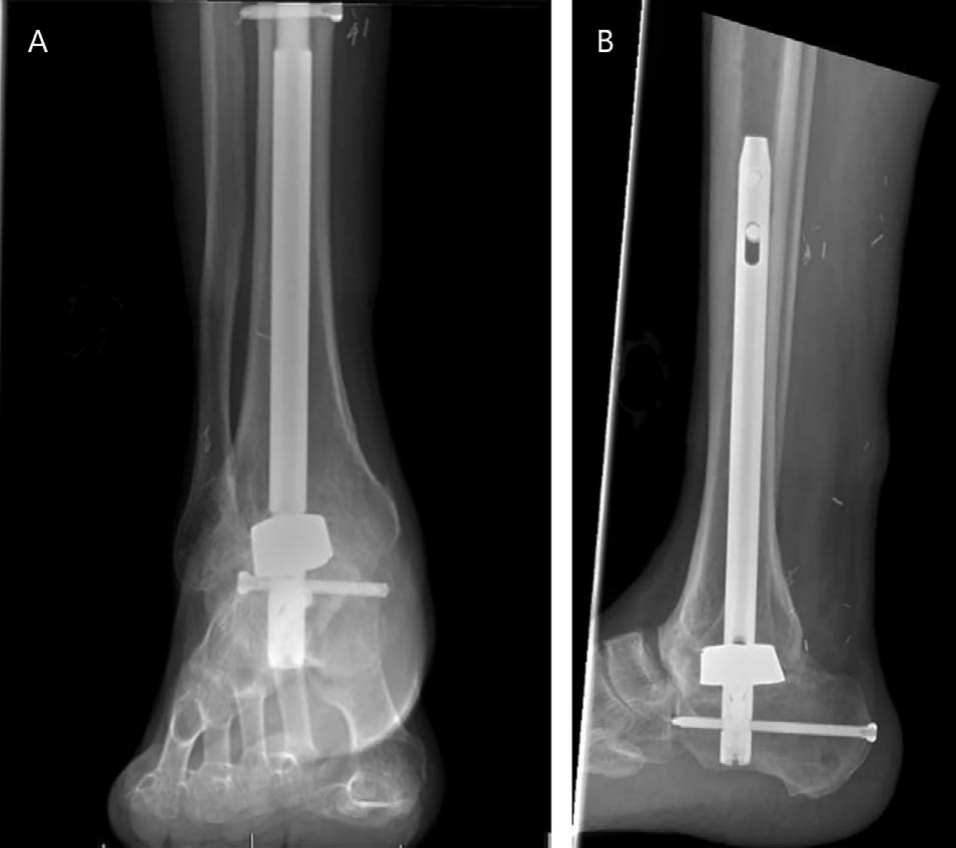
(A) Anteroposterior and (B) lateral x-rays of the patient's right ankle 2 years after hindfoot fusion show complete fusion of the tibia and calcaneus.

The patient was discharged non-weight-bearing for 6 weeks. He transitioned to partial weight-bearing in a walking boot with an increase of 30% weight-bearing every week. At 6-week follow-up, dehiscence of a small aspect of the anterolateral incision was found, and the patient was placed on 500 mg cefalexin 4 times daily for 6 weeks. His wound healed 3 months postoperatively, and use of the walking boot was discontinued. The patient was able to ambulate without any assistive devices. The patient reported minimal to mild intermittent ankle pain for the next 2 years after his surgery. At 2-year follow-up, the patient's fusion had healed well without any signs of nonunion (Figure 5).

## DISCUSSION

Talar extrusion results either from excessive supination or excessive pronation with high-energy trauma.^2^ Complete talar extrusion is commonly associated with avascular necrosis, osteoarthritis, limited range of motion, and infection.^1,14-18^ Treatment outcomes can be highly variable with high complication rates.^1^ The goals of treatment are to restore functionality to the ankle and limit complications.^1^

Reimplantation, open reduction and internal fixation, and tibiocalcaneal arthrodesis are the most common treatment options for talus extrusion.^1,8,14,15,18^ If the talus is viable, reimplantation is recommended.^1,8,15,16^ In a retrospective study, Smith et al reported that reimplantation had a low rate of infection, maintained flexibility, and preserved the normal ankle anatomy as much as possible.^15^ Because of complications such as avascular necrosis and infection after these injuries, some surgeons have performed a talectomy with a tibiocalcaneal arthrodesis even when the talus is present.^1,19^ However, a systematic review of total talus dislocation by Weston et al showed that talectomy did not decrease the risk of infection compared to cases where the talus was preserved.^1^ Weston et al reported a 10% incidence of infection in cases of talectomy and reimplantation.^1^ Consensus is limited on the definitive treatment when the talus is not recovered after initial trauma.

Tibiocalcaneal arthrodesis has been shown to improve outcomes in patients who undergo primary talectomy.^1,5,9-11,18-20^ A few cases suggest that tibiocalcaneal arthrodesis completed with retrograde intramedullary nail may be a definitive treatment for cases of extruded talus.^11-13^ Cuervas-Mons et al describe a case of an extruded talus treated with retrograde tibial nail and trabecular titanium spacer block that prevented leg length dysmetria and provided good function in the ankle overall.^11^

However, the search for a standard treatment for extruded talus continues, as many cases show positive outcomes with different interventions. Some studies show reasonable outcomes in patients undergoing tibiocalcaneal arthrodesis with no spacer.^12,13,16,18,20^ Other patients whose talus is missing have had good outcomes with tibiocalcaneal arthrodesis with a talar body prosthesis or autograft.^7,9-11,21^ These cases suggest that stability of the ankle and restoration of hindfoot height is best preserved with a tibiocalcaneal arthrodesis involving a spacer to fill the void previously occupied by the talus. Amputation can be considered when the talus is not viable or not present.

## CONCLUSION

For our patient, placement of an external fixator and antibiotic cement spacer was complicated by limited range of motion, pain, reduction in ambulation, and recurring infections. Because of our patient's recurrent ankle infections and chronic instability from the missing talus, the tantalum cone was selected as a spacer and autologous bone grafting was obtained via reamer irrigator aspirator to provide more stability to the joint and prevent leg length discrepancy. The use of a tantalum metal spacer (available in total knee arthroplasty sets), the reamer irrigator aspirator, and the chronicity of the injury make this case unique. Our surgical approach and technique led to a good outcome for our patient after a morbid injury, allowing him to ambulate with minimal ankle pain without assistive devices and avoiding the need for an amputation. However, given the rarity of this injury and the short follow-up for many of these cases, research with long-term follow-up is needed.

## Notes

*This article meets the Accreditation Council for Graduate Medical Education and the American Board of Medical Specialties Maintenance of Certification competencies for Patient Care and Medical Knowledge.*
